# Traditional Eddy Current–Pulsed Eddy Current Fusion Diagnostic Technique for Multiple Micro-Cracks in Metals

**DOI:** 10.3390/s18092909

**Published:** 2018-09-01

**Authors:** Zhenwei Wang, Yating Yu

**Affiliations:** 1 School of Aeronautics and Astronautics, University of Electronic Science and Technology of China, Chengdu 611731, China; 2 School of Mechanical and Electrical Engineering, University of Electronic Science and Technology of China, Chengdu 611731, China; wzwyyt@uestc.edu.cn

**Keywords:** metals, multiple micro-cracks, nondestructive techniques fusion, pulsed eddy current (PEC), traditional eddy current (TEC)

## Abstract

Due to a harsh working environment, micro-cracks in metal structures (e.g., airplane, oil/gas pipeline, hydro-turbine) often lead to serious accidents, so health monitoring of the metals is of great significance to ensure their safe operation. However, it is hard to perform quantitative detection of multiple micro-cracks by a single nondestructive testing (NDT) technique because of their limits. To monitor for multiple micro-cracks in metals, a Traditional Eddy Current (TEC) and Pulsed Eddy Current (PEC) fusion NDT technique is proposed in this paper. In the proposed technique, the TEC technique is adopted to seek the locations of the micro-cracks in the whole of the metal, while the PEC technique is adopted to acquire information on the depth of micro-cracks automatically according to the location information by the TEC. The experiments indicate that the TEC–PEC fusion NDT system can localize the micro-cracks as well as detect the micro-cracks quantitatively and automatically; therefore, it can be applied in structural health monitoring of metal equipment or in picking candidate components in re-manufacturing engineering.

## 1. Introduction

Due to a harsh working environment, micro-cracks in large metal equipment often result in serious accidents, so health monitoring of the key components of metal equipment is of great significance. Nondestructive Testing (NDT) technology is one of the effective methods for structural health monitoring. It is applied for quality control (QC)/quality assurance (QA) and inspection of critical components in the aerospace, railroad, transportation, and energy plant sectors. For example, the pulsed eddy current (PEC) technology has a unique advantage in the quantitative detection of micro-cracks because of its rich spectrum and fast response [1,2,3,4].

At present, most of the research for micro-crack detection is focused on single micro-cracks [5,6,7,8,9]; however, multiple micro-cracks usually occur in the same component, so more attention should be paid to multiple micro-cracks in metals. Gao et al. [10] present a novel unsupervised sparse component extraction algorithm to identify micro-cracks and their locations by an eddy current pulsed infrared thermography imaging system, and the proposed method has significantly improved the accuracy of defect detection by 60% in terms of the F-score.

It is well-known that different NDT techniques exhibit different strengths and limitations. Given the variety of multiple cracks, it is often necessary to employ more than one NDT technique. When employing two or more methods and combining their data sets, a unified representation is generated that describes different aspects of the metals at once and thus offers simplified interpretability; this is called an NDT fusion.

In some cases, sources of information on the same aspect of an object are fused to reduce uncertainty and thus achieve increased detection robustness and accuracy [11,12]. In some cases, multi-sensor NDT is capable of combining complementary data, enhancing the signal-to-noise ratio (SNR), and performing more accurate defect detection [13,14], while in some cases multi-NDT technologies are fused to obtain more information about the metal structures with high detection efficiency [15,16,17]. Some efforts in this direction have been made in mostly academic settings.

In this paper, to detect multiple micro-cracks in metals, the traditional eddy current (TEC) and pulsed eddy current (PEC) techniques are fused to obtain information on the locations and geometries of micro-cracks in metal structures by an experimental investigation. The complementary information from TEC and PEC makes micro-cracks detection rapid and efficient.

The rest of the paper is organized as follows. An approach to measure the locations of multiple micro-cracks in metal is proposed based on TEC; then, research on a detection approach to micro-cracks by PEC is presented. Then, the fusion NDT approach for multiple micro-cracks detection in a metal component based on TEC and PEC is presented, and a quantitative and automatic detection platform for multiple micro-cracks is set up based on the fusion approach and some experiments are carried out. Finally, some conclusions are drawn.

## 2. Sample Preparation

The sample with the micro-cracks mentioned in this paper was manufactured by the wire electrical discharge machining (WEDM) process. The material is aluminum alloy 7075, which is commonly used in aerospace engineering. The conductivity of the sample σ is 26.77 MS/m, and the relative magnetic permeability *μ*_r_ is 1. The width of cracks is 0.3 mm, which is the minimal size processed by WEDM. The width of the cracks is much smaller than those in existing investigations of PEC crack characterization [3,4], so we called the cracks in the paper micro-cracks.

**Figure 1:**
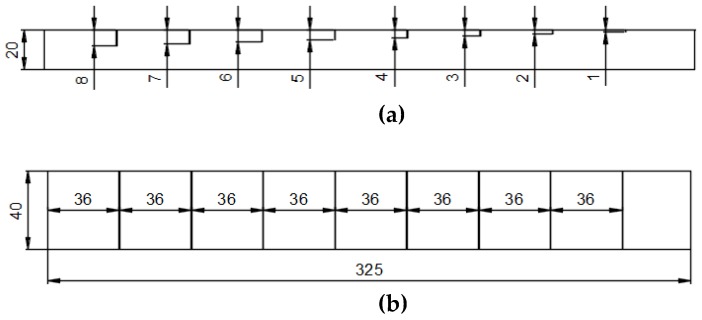
The geometric parameters of the sample: (**a**) front view; (**b**) top view (unit: mm).

The length of the micro-cracks is 40 mm, and their depth varies from 1 mm to 8 mm with a step of 1 mm considering the skin depth. In PEC, the standard skin depth *δ*_1_ can be calculated according Equation (1) as 3.1 mm. According to [18], the maximum depth of a crack that can be detected by the PEC is 4*δ*_1_. The geometric parameters of the sample are shown in Figure 1.

(1) $$\delta_{1}=\sqrt{\frac{2}{\omega_{1}\sigma \mu }}=\frac{1}{\sqrt{\pi f_{1}\sigma \mu }}$$

where *δ*_1_ is the standard skin depth, *μ* = *μ*_0_*μ*_r_ and *μ*_0_ = 4π × 10^−7^ H/m, and *ω*_1_ = 2π*f*_1_ is the fundamental angular frequency of the excitation signal.

## 3. Traditional Eddy Current Technique to Locate Multiple Micro-Cracks

### 3.1. Working Principle of Traditional Eddy Current Technique

For the eddy current testing technique, an alternating current *I*_1_ in the driving coil creates an alternating magnetic field H_1_, which is the primary magnetic field and induces current *I*_2_ in the sample. The eddy currents simultaneously generate a secondary magnetic field H_2_, which resists the variation of the primary magnetic field and changes the resultant magnetic field H. H is dependent on such factors as the lift-off *l*, the excitation frequency *f*, the sample’s electrical conductivity σ, the sample’s relative magnetic permeability *μ*_r_, and probe coil geometry parameters (the inner radius *r*_1_, the outer radius *r*_2_, height *h*, and the number of turns *N*). The Z-component of the magnetic flux density *B*_z_ is commonly used as the detection signal because it is strong enough to be detected by a solid magnetic sensor (e.g., a Hall sensor, a GMR sensor, or a TMR sensor). Therefore, *B*_z_ can be expressed as

(2) $$B_{z}\sim (N,I_{1},r_{1},r_{2},h,l,f,\sigma ,\mu_{r}).$$

### 3.2. Experiments and Result Analysis

**Figure 2:**
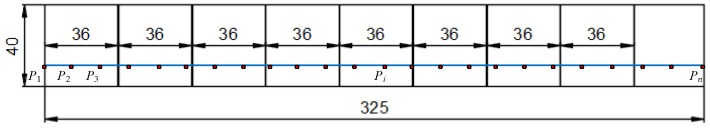
The distribution of the detection point along the sample.

**Figure 3:**
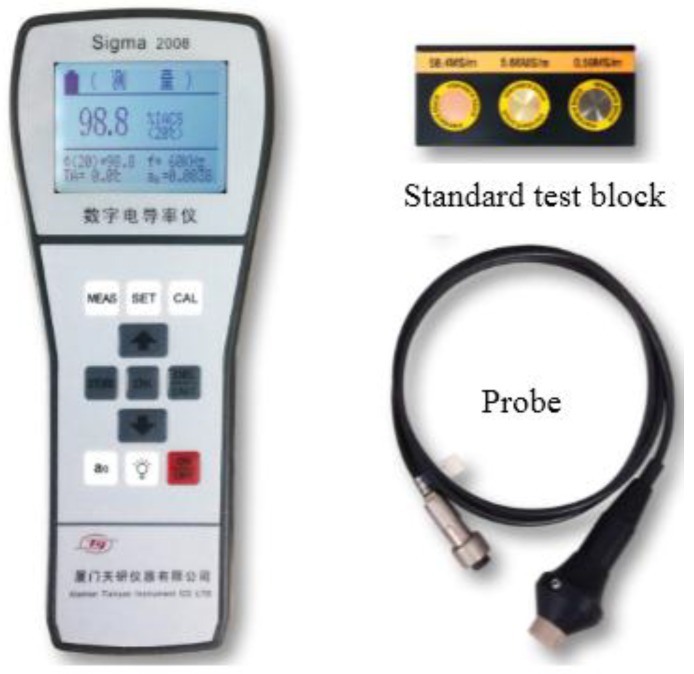
The sigma 2008 digital conductivity meter.

**Figure 4:**
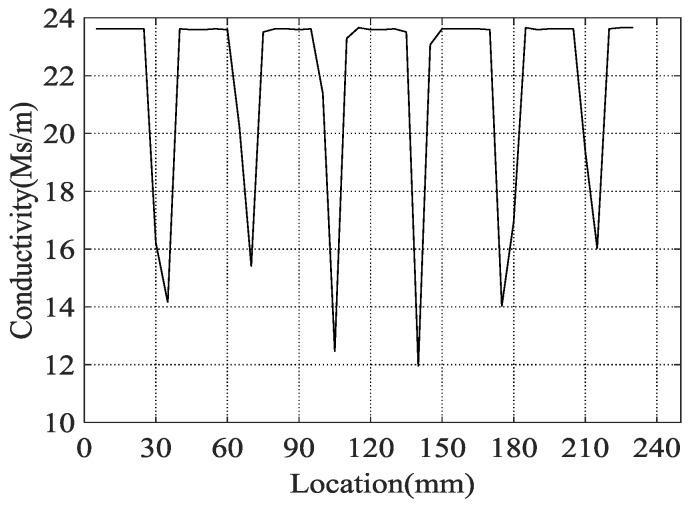
The conductivity of the sample along the sample.

In the experiment, the conductivity at points *P*_1_, *P*_2_, …, *P_i_*, …, and *P_n_* (Figure 2) are detected by the sigma 2008 digital conductivity meter (Figure 3), which is a conductivity meter designed according to the traditional eddy current working principle. Before the experiment, the sigma 2008 digital conductivity meter was calibrated by standard test blocks. In the experiment, the excitation frequency was 60 KHz, and the interval of the adjacent detection points was equal to 5 mm. The conductivities at the detection points are detected and plotted in Figure 4. From Figure 4, we can see that the micro-cracks have an obvious influence on the conductivity of the detection points. When the probe of the sigma 2008 digital conductivity meter approaches the micro-crack, the eddy current generated in the sample is seriously disturbed by the micro-crack because the conductivity of the micro-crack is much less than the conductivity of the sample. The closer the detection point is to the micro-crack, the stronger the influence of the micro-crack on the eddy current in the sample. Therefore, the peak value of the relationship between the conductivity along the sample can characterize the location of the micro-cracks.

Therefore, we acquired the locations of the peak values and list them in Table 1. In Table 1, L_r_ is the calibration location of micro-cracks, L_m_ represents the location characterized by the sigma 2008 digital conductivity meter, and E_a_ and E_r_ are the absolute error and relative error, respectively.

**Figure 5:**
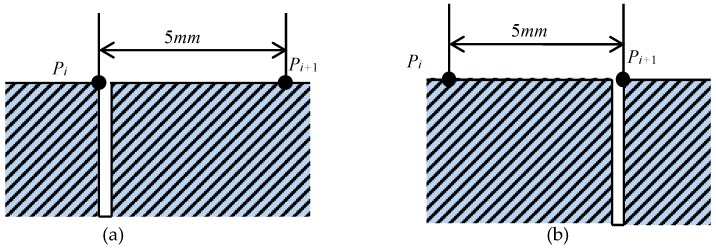
Two extreme cases of detection point *P_i_* and *P_i_*_+1_: (**a**) *P_i_* located at the left wall of the micro-crack; (**b**) *P_i_*_+1_ located at the right wall of the micro-crack.

According to Table 1, we can see that the locations of the peak values of the conductivity of the sample at the different locations are much closer to their calibrated locations. The maximal absolute error is 5.75 mm at micro-crack 5, while the maximal relative error is 3.18%. The error comes from two aspects: one is the systematic error in the detection, the other is a manufacturing error in the wire electrical discharge machining process during the sample’s manufacture. In our experiment, the systematic error dominates. The systematic error can be explained as follows: in the experiment, two extreme cases of the detection point and the micro-crack show up, which are described in Figure 5.

For case (a), the peak value appears at *P_i_*, while for case (b), the peak value appears at *P_i_*_+1_; therefore, the maximal systematic error is the interval of the adjacent detection points (5 mm in this paper). Therefore, the smaller the interval of the adjacent detection points, the more accurate the characterizations by the sigma 2008 digital conductivity meter. A smaller interval of the adjacent detection points means that more detection points and more conductivities should be measured, which will lower the localization efficiency.

## 4. Pulsed Eddy Current Technique to Quantitatively Characterize Micro-Cracks

### 4.1. PEC Experimental Setup

**Figure 6:**
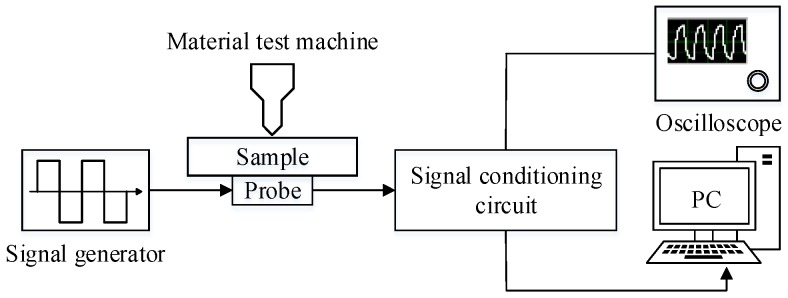
The module diagram of the pulsed eddy current (PEC) testing platform. PC, personal computer.

The PEC experimental testing platform for characterization of the micro-cracks in this paper consists of an excitation source, a probe (including one coil and one magnetic Hall sensor), the sample, a signal-conditioning circuit, a data acquisition card, and a computer with signal-processing software. The module diagram and the hardware of the PEC testing system are shown in Figure 6.

In Figure 6, the function generator generates a square wave signal. The square wave signal can be expressed as the sum of odd harmonic signals according to the Fourier series principle. When the duty ratio is 50%, the square wave signal used in the experiment can be extended as (3).

(3) $$I_{p}(t)=\frac{a_{0}^{2}}{\pi }\left(\sin2\pi ft+\frac{1}{3}\sin3\cdot 2\pi f+\frac{1}{3}\sin5\cdot 2\pi f+\frac{1}{3}\sin7\cdot 2\pi f+\cdots \right)$$

where *a*_0_ is the amplitude and *f* is the excitation frequency of the square wave signal. Because the square wave signal contains rich information in the frequency domain, it is commonly used in crack characterization [1,2,6].

The detection module is packaged by an excitation coil and a hall sensor, which is fixed to a high-precision displacement calibrator and is placed over the micro-cracks. The hall sensors in the probe can measure the magnetic electromagnetic signals related to a micro-crack. An amplification filter circuit is designed to amplify the weak signal from the hall sensor and to filter the noise signal so that the characteristic signal can be effectively identified. Then, the conditioned signal was input to the data acquisition card and further processed by the signal-processing program embodied in the host computer.

### 4.2. Experimental Results

**Figure 7:**
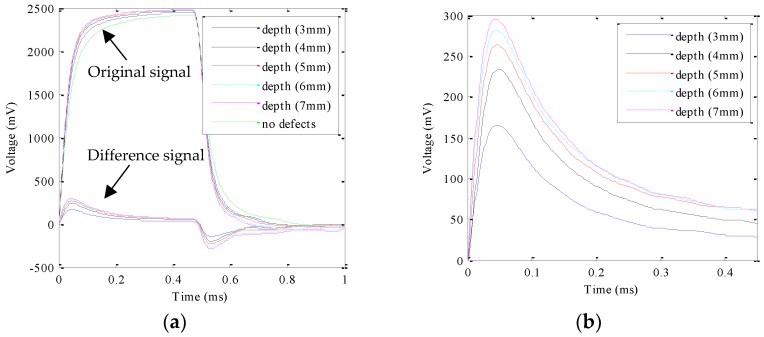
Time domain signal of PEC experiments: (**a**) whole period; (**b**) the differential signal from 0 ms to 0.45 ms.

In the PEC experiments, a square wave signal with the frequency of 1 kHz, the amplitude of 12 V, and the duty ratio of 50% was determined as the excitation signal. The height, the inner diameter, and the outer diameter of the excitation coil is 2 mm, 7 mm, and 15 mm, respectively. The detection magnetic sensors used were UGN3503 Hall sensors. The sample was prepared as shown in Figure 1. It is worthwhile mentioning that the PEC probe is located right above the micro-cracks in the experiment in this section. The detected original signals and the differential signals (the signal of no micro-crack is the reference signal) for each micro-crack were obtained and are shown in Figure 7a. When the crack depth is 8 mm, the original detection signal is almost overlapped by the signal at the crack depth of 7 mm because the crack depth is bigger than the skin depth. In order to show the experimental signal clearly, the experimental data at the crack depth of 8 mm was removed. To observe the peak value of the differential signal at the rising edge of the square wave, the experimental data from 0 ms to 0.45 ms was extracted and are shown in Figure 7b.

As can be seen in Figure 7, the peak value increases with increasing micro-crack depth. Besides this, the peak time keeps constant after 0.046 ms for each micro-crack. Therefore, the peak value of the voltage from the Hall sensor can be used to characterize the depth of the micro-crack. The relationship between the peak value and the micro-crack depth can be expressed as

(4) $$D_{m}=2.77\times 10^{-4}\cdot V_{\mathrm{peak}}{}^{2}-9.76\times 10^{-2}\cdot V_{\mathrm{peak}}+11.58$$

Table 2 lists the characterization depth of micro-cracks according to Equation (4). In Table 2, D_c_ is the calibration depth of a micro-crack, *V*_peak_ is the peak value of the voltage signal from the Hall sensor, D_m_ is the characterization depth of a micro-crack, and E_a_ and E_r_ are the absolute error and the relative error, respectively.

For micro-crack depth characterization, as shown in Table 2, the maximal absolute error is 0.10 and the maximal relative error is 2.50%, which indicates that PEC can quantitatively detect the depth of micro-cracks with high accuracy.

## 5. Fusion NDT Technique Based on TEC and PEC

### 5.1. Fusion Strategy

According to the analysis in Section 3 and Section 4, TEC can locate micro-cracks in large-scale metal structures rapidly and conveniently, and PEC can quantitatively characterize the depth of a micro-crack. To detect multiple micro-cracks in metals, TEC and PEC can be fused, in which TEC locates multiple micro-cracks, and PEC detects the depth of micro-cracks according to information on their location from TEC.

**Figure 8:**
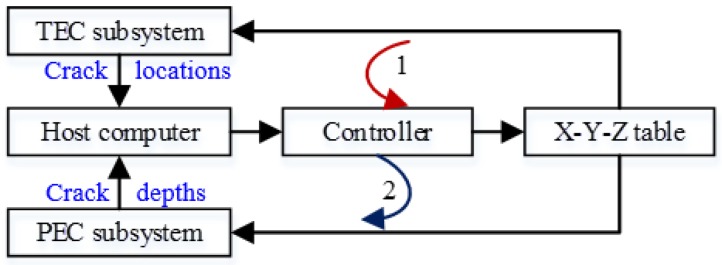
Fusion strategy of TEC and PEC.

The fusion strategy of TEC and PEC is shown in Figure 8. The TEC–PEC fused NDT system works as follows: First, the sample is scanned by the TEC probe with the help of the X-Y-Z table controlled by the host computer, and the detection signal from TEC is sent to the host computer to determine the micro-cracks’ locations; then, information on the location of the micro-cracks is sent to the motion control card and drives the X-Y-Z table, where the probe of the PEC system is fixed on, to move to the first micro-crack location. After that, the PEC system is activated, the quantitative detection of micro-cracks is carried out, and the micro-crack depth is transmitted to the computer. After 10 s, the X-Y-Z table fixed to the probe of the PEC system starts and moves to the next micro-crack until the last crack has been detected by the PEC subsystem.

**Figure 9:**
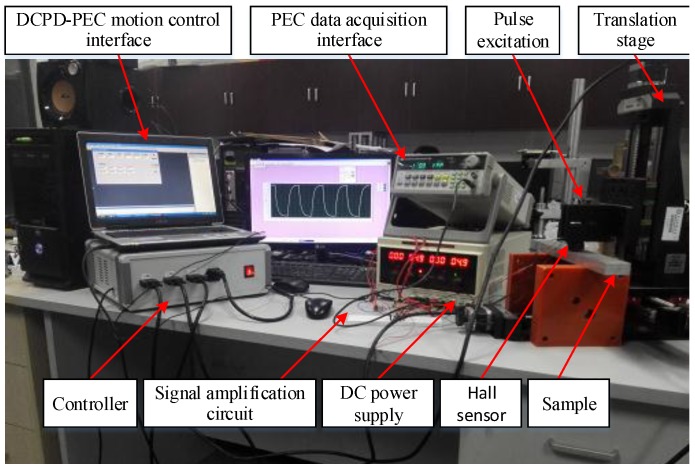
The hardware of TEC–PEC fused system. DC, direct current.

According to the fusion strategy of TEC and PEC shown in Figure 8, the interface program between the TEC subsystem and the PEC subsystem is coded to realize the data transmission, and then the automatic detection for the locations and the depths of multiple micro-cracks in metals can be realized. The hardware of the TEC–PEC fused testing system is shown in Figure 9.

**Figure 10:**
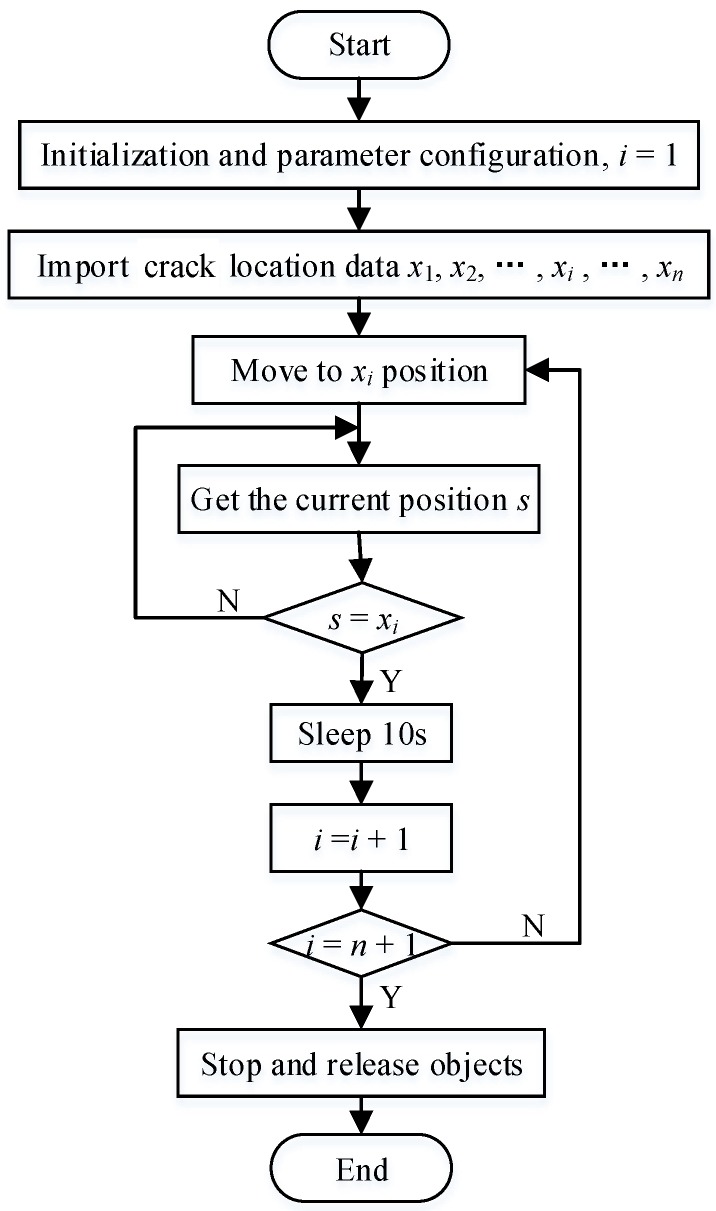
Motion control program flow chart.

In the fused NDT system, a 9030 motion control card and a ZP140-200 X-Y-Z table were used to link the TEC subsystem and the PEC subsystem. On the one hand, the 9030 control card connects the ZP140-200 X-Y-Z table through an RS232 serial port, and it controls the movement of the PEC probe fixed on the ZP140-200 X-Y-Z table. On the other hand, the 9030 control card is controlled by the host computer through VB coding according to the location data from TEC. The motion control program flow chart is shown in Figure 10.

### 5.2. Experimental Results and Analysis

**Figure 11:**
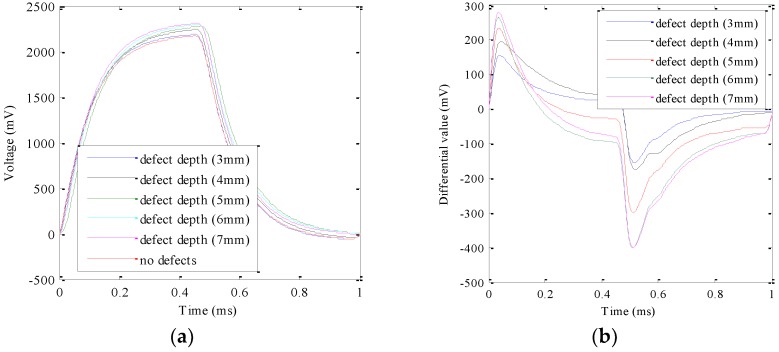
The original signal (**a**) and differential signal (**b**) of the fused nondestructive testing (NDT) technique by experiment.

The experimental parameters in the TEC–PEC fused NDT testing system were the same as those in Section 3 and Section 4. The detected signals with different depths and their differential signals were obtained and are shown in Figure 11. Comparing Figure 7 and Figure 11, we can see that the TEC–PEC fused NDT technique can detect multiple cracks as well as the PEC subsystem. However, the different signals exhibit obvious intersections. This error comes from the location error in the TEC subsystem, which is shown in Table 2. The relationship of the peak values of the differential signals at the different crack depths are expressed as (5).

D_f_ = −4.36*V*_peak_^2^ + 75.73 *V*_peak_ − 34.71 (5)

where *V*_peak_ is the peak value of the voltage signal from the Hall sensor and D_f_ is the crack depth by the fused NDT technique. According to (4), we can obtain the different crack depths, which are listed in Table 3. In Table 3, the errors of the TEC–PEC fused testing platform from the experiments are also listed.

In Table 3, E_ad_ and E_rd_ are the absolute and relative error, respectively. As can be seen from Table 2, the maximal absolute error of the fusion NDT system is 0.13 mm when the crack depth is 6 mm, and the maximal relative error is 2.5% when the crack depth is 4 mm. The location error by TEC is the main reason for error in the fused NDT technique.

The experimental result indicates that the TEC–PEC fused NDT technique can locate and quantitatively characterize micro-cracks in the metal structure at one time with high accuracy. In the TEC–PEC fused NDT technique, the crack locations detected by TEC can be utilized to automatically locate the probe of the pulsed eddy current technique, which can quantitatively detect the micro-cracks; therefore, automatic detection for micro-cracks in metals can be carried out.

## 6. Conclusions

For key metal structures, a micro-crack’s location and its depth are the parameters that draw the most concern. This paper proposed a TEC–PEC NDT fusion technique for automatic and quantitative detection of multiple micro-cracks in metals. Firstly, the capabilities of TEC in locating micro-cracks and PEC in quantitatively characterizing the depth of the micro-cracks are investigated by experiment. Then, a fusion strategy for TEC and PEC was presented and the TEC–PEC fusion platform was designed. Based on the fusion platform, some experiments were carried out. Some conclusions can be drawn:

(1) Multiple micro-cracks can be located by TEC because the location of the peak signal corresponds to the micro-cracks’ locations.

(2) Micro-crack (manufactured by the wire electrical discharge machining process) quantitative detection can be performed by PEC because the peak signal is quadratic with the depth of micro-cracks.

(3) In the proposed TEC and PEC fused NDT system, the probe of the PEC subsystem can be located over the micro-cracks according to information from the TEC subsystem; therefore, multiple micro-cracks in metals can be detected automatically and quantitatively by the proposed TEC and PEC fused NDT system with high accuracy.

The investigations in this paper not only indicate that TEC has high performance in micro-crack location and that PEC has a strong capability for evaluating the micro-cracks’ depth, but also indicate that the TEC–PEC fused technique can realize fast and accurate detection of multiple micro-cracks in metals at one time. The information from the TEC subsystem and that from the PEC subsystem are complementary, so the TEC–PEC fused diagnostic technique can be readily applied in health monitoring for large-scale metal structures or in picking candidate components in re-manufacturing engineering.

## Figures and Tables

**Table 1:** Micro-crack locations by traditional eddy current (TEC) and errors.

Microcrack #	Depth (mm)	L_r_ (mm)	L_m_ (mm)	E_a_ (mm)	E_r_ (%)
1	8	36.15	35	1.15	3.18
2	7	72.3	70	2.3	3.18
3	6	108.4	105	3.45	3.18
4	5	144.6	140	4.6	3.18
5	4	180.7	175	5.75	3.18
6	3	216.9	215	1.9	0.88
7	2	253.0	250	3.05	1.20
8	1	289.2	285	4.2	1.45

**Table 2:** Experimental Results and Errors.

D_c_ (mm)	3	4	5	6	7
*V*_peak_ (mV)	165.1	233.7	263.5	281.9	295.2
D_m_ (mm)	3.01	3.90	5.09	6.07	6.90
E_a_	0.01	0.10	0.09	0.07	0.09
E_r_ (%)	0.33	2.50	1.80	1.17	1.29

**Table 3:** Results and errors of the TEC–PEC fusion system.

D_c_ (mm)	3	4	5	6	7
L_m_ (mm)	105	140	175	215	250
D_f_ (mm)	3.04	3.90	5.02	6.13	6.88
E_ad_ (mm)	0.04	0.10	0.02	0.13	0.12
E_rd_ (%)	1.33	2.5	0.4	2.16	1.71
